# Longitudinal changes in hand dominance and manual dexterity in normal aging and mild cognitive impairment (MCI): a kinematic study

**DOI:** 10.3389/fnagi.2026.1921208

**Published:** 2026-10-02

**Authors:** Olena Vasylenko, Claudia Rodríguez-Aranda

**Affiliations:** Department of Psychology, UiT The Arctic University of Norway, Tromsø, Norway

**Keywords:** manual asymmetry, manual dexterity, mild cognitive impairment—MCI, normal aging, Purdue Pegboard Test, symmetric hand dexterity

## Abstract

**Introduction:**

Decrements on hand dominance in older populations, including neurodegenerative conditions, have been reported cross-sectionally. At present, no longitudinal research on this issue is available.

**Methods:**

To address this research gap, we conducted a detailed kinematic analysis of unimanual movements of the dominant and non-dominant hands in 16 patients with mild cognitive impairment (MCI) (M age = 69.3 yrs) and 20 healthy controls (HC) (M age = 69.9 yrs) at two points in time with an averaged time interval of 4.5 years between evaluations (3.5 yrs. for HC; 5.35 yrs. for MCI). The inserting pins task from the Purdue Pegboard Test was recorded with a 2D motion capture system (Vicon Motus). Linear and angular velocities, as well as path length together with their coefficients of variation were calculated for specific actions (i.e., reaching, grasping, transport and inserting) and laterality indices (LI) were computed.

**Results:**

At time 1, only MCI patients displayed symmetric movements. At time 2, no group differences in LIs were observed as the HC group also developed symmetric hand dominance while MCIs preserved symmetric hand dominance. The present study is the first one to demonstrate longitudinally the presence and development of reduced manual asymmetry in two groups of older adults with and without cognitive impairment.

**Discussion:**

These findings are fundamental to document the development of symmetric hand performance in old age, which most likely represents a behavioral adaptation to normal neurophysiological constraints caused by the wear and tear of the neuromuscular system, rather than being solely the result of pathology. However, conditions like MCI that are superimposed upon normal senescence processes appear to accelerate the development of reduced manual asymmetry.

## 1 Introduction

Age-related lateralized changes in brain and behavior have been reported in a vast number of reviews related to healthy aging and dementia (e.g., Keller et al., 2019; Tagliabue et al., 2022). An example of such phenomenon is the reduction in cerebral lateralization in older people, which has been conceptualized by the neurocognitive model of HAROLD, i.e., Hemispheric Asymmetry Reduction in Older Adults (Cabeza, 2002). This framework posits that an increase in bilateral cerebral activation occurs in aging as a result of compensatory mechanisms vis-à-vis normal neurocognitive decline. In parallel, the behavioral manifestation of this bilateral reorganization has also been evaluated through handedness, which is a well-established asymmetric pattern of motor function (Gooderham and Bryden, 2014). Handedness is defined as the preferential use of one hand over the other and it is established early in life (Andersen and Siebner, 2018). Because handedness is a strong lateralized motor preference and thus, a manifestation of brain lateralization, it has been explored in connection with the age-related reduction in functional asymmetry in Alzheimer’s disease (De Leon et al., 1986), Parkinson’s disease (Uitti et al., 2005), amyotrophic lateral sclerosis (Turner et al., 2011) and cognitively healthy older adults (Przybyla et al., 2011).

The clinical interest in studying handedness in older adults relates to the phenomenon that many neurodegenerative diseases associated with aging show initial pathological changes on one side of the body (Lubben et al., 2021). Actually, handedness has been linked to different dementia types (Mundorf et al., 2025) and even proposed as a marker of neurodegeneration (Wiberg et al., 2019). However, recent reviews based on meta-analytical approaches have failed to reach conclusions as to the association between handedness and dementia (Abuduaini et al., 2024; Mundorf et al., 2025). There are many reasons for the inconclusive findings of these reviews. On one hand, there are huge variations in the definitions and methods used to evaluate handedness across studies, which range from subjective self-reports to established inventories with varying cut-off scores (Mundorf et al., 2025). On the other hand, the scarce research applying objective methods to evaluate hand function has been conducted at a single point in time (e.g., Ilardi et al., 2025). This brings us to the next constraint, namely that disregarding the methods adopted, research dealing with handedness and aging is based on cross-sectional studies. As such, findings derived cross-sectionally only inform about cohort differences at the time of testing and cannot determine the progressive effects of aging or age-related diseases.

To move forward on this line of investigation, various paths of action need to be followed. First, rather than using questionnaires to measure handedness, it would be appropriate to focus on the evaluation of hand dominance through objective assessment of hand function. Second, it is important to answer what the role of aging is in connection with changes of hand dominance in older adults with and without pathology. Third, only a longitudinal evaluation will clarify whether changes of hand dominance are linked to pathology or not. Based on the above considerations, the present investigation aims to evaluate changes in hand dominance through a detailed kinematic analysis of unimanual movements of the dominant and non-dominant hands. Two groups of right-handed older adults are evaluated. One of them comprises healthy individuals and the other, patients diagnosed with mild cognitive impairment (MCI). The rationale to focus on these specific groups relies on the effort of examining age-related changes from an aging continuum approach. Healthy controls and MCI patients represent two stages of the aging continuum that are tested after a period of approximately 4.5 years from initial evaluation. Cross-sectional results of these groups already reported (Vasylenko et al., 2022) demonstrated a loss of hand dominance in MCI patients, and typical manual asymmetries proper of right-handed individuals in healthy controls. These results also showed a deterioration of fine manual dexterity in MCI patients compared to healthy controls. In the present longitudinal evaluation, we expect that the MCI group will preserve symmetric hand dominance and more deteriorated hand movements. For the control group, we rely on the working hypothesis that normal aging causes detrimental effects on hand dexterity (e.g., Marmon et al., 2011) and as such, we expect that healthy older participants also show deteriorated hand function in terms of slower and less precise movements. However, we refrain from proposing specific hypotheses about developmental trajectories in manual asymmetry of healthy participants due to the mixed results of studies showing both unchanged (Francis and Spirduso, 2000; Gooderham and Bryden, 2014; Teixeira, 2008) and reduced hand dominance (Pan et al., 2023; Przybyla et al., 2011; Sebastjan et al., 2017) in normal aging.

## 2 Materials and methods

In the following section we will provide thorough information about the participants (section 2.1) and their assessments, including testing times and inclusion criteria for the study. Under section 2.2 we present the instrumentation and methodology employed for acquisition and processing of kinematic data as well as for the calculation of laterality indices. Finally, sections 2.3 and 2.4, respectively, describe the general procedures for the testing sessions and the rationale for the statistical analyses.

### 2.1 Participants

The present study belongs to a major longitudinal umbrella project investigating the association of various motor functions and cognition in older adults with and without cognitive decline. The project was approved by the Regional Research Ethics Committee (Ref. REK 2009/1427) and carried out in accordance with the Helsinki guidelines. A total of 36 individuals from the study reported in Vasylenko et al. (2022) successfully underwent a second evaluation ~4.5 years apart with complete dataset. This sample encompassed 16 MCI patients, 11 female, M (SD) age_Time1_ = 69.3 yrs. (6.60), M (SD) age_Time2_ = 74.6 yrs. (6.37) and 20 healthy controls (HC), 6 female, M (SD)age _Time1_ = 69.9 yrs. (3.92), M (SD) age_Time2_ = 73.5 yrs. (3.89). The patient group was diagnosed at Time 1 (T1) with F06.7 Mild cognitive disorder in agreement with the ICD-10 guidelines by specialists from the Department of Geriatrics and Neurology at the University Hospital of North Norway (UNN). At Time 2, the patient group was conformed only by individuals who presented unchanged cognitive functioning based on a complete neuropsychological battery that was applied at both assessment times. Hence, the patients evaluated at retest time represent a stable MCI group. The HC group underwent the same neuropsychological assessments as patients in both assessment times to ensure that they were cognitively healthy. A complete description of the assessments has been reported elsewhere (Gorecka et al., 2021). Figure 1 shows the number of participants at both times of assessments on each group as well as the reasons for dropout.

**Figure 1:**
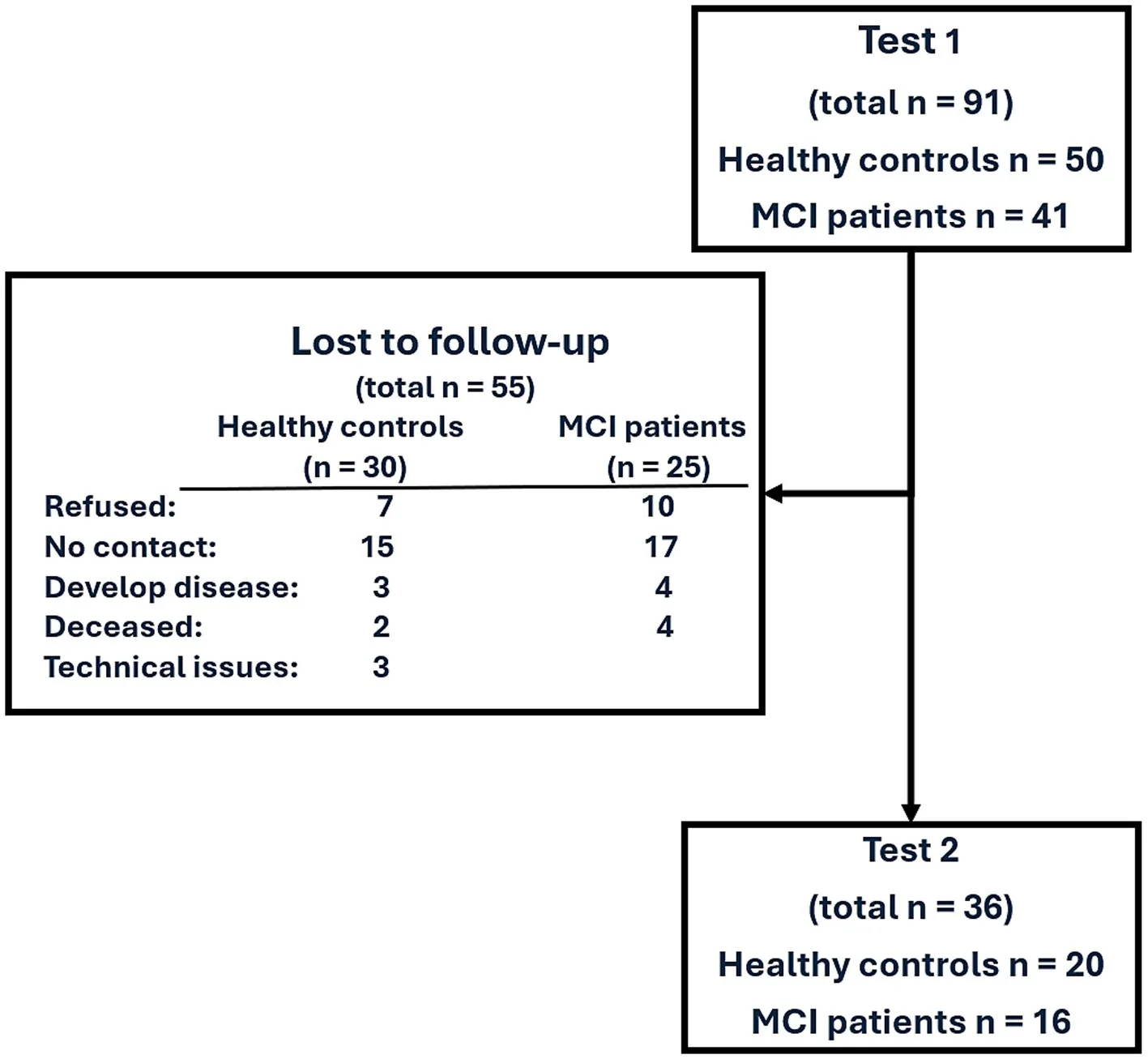
Flow diagram of participation in the study showing reasons for dropout between Time 1 and Time 2 of assessments.

Evaluation of the groups occurred at different points in time. The first evaluation (T1) took place between 2014 and 2016 for the HC group, and between 2017 and 2020 for the MCI group. The second evaluation (Time 2, T2) occurred between 2017 and 2021 for HCs, while MCI patients were retested between 2021 and 2025. The mean time for the follow-up assessment was 3.5 (SD = 1.10) years for HCs and 5.35 (SD = 0.81) years for patients. At T2 all participants received information about the study and gave written informed consent before testing. Participants completed an interview to obtain demographic and health information. Additionally, several screening measures were administered including the Briggs–Nebes Handedness Inventory (Briggs and Nebes, 1975) to assess hand preference and the Mini-Mental State Examination (MMSE) (Folstein et al., 1975) to assess global cognitive status.

Inclusion criteria were ability to consent and capacity to move the hands. Consent capacity in the patient group was carefully evaluated on a case-by-case basis by telephone conference and relying on the UCSD Brief Assessment of Consent Capacity (UBACC) questionnaire (Jeste et al., 2007). Exclusion criteria encompassed pathology that directly affected the musculoskeletal system (i.e., rheumatoid arthritis, multiple sclerosis, Parkinson’s disease), eyesight problems, acute illness, current hand pain, and arm or hand injuries around testing time. For the HC group, an additional criterion for exclusion was an MMSE score of ≤27.

### 2.2 Kinematic measures and laterality indices

Two subtests of the Purdue Pegboard Test (PPT) (Tiffin, 1968) were selected for this study: inserting pins with the right (dominant) hand and inserting pins with the left (non-dominant) hand. The tasks were given in that order, and each task required the participants to insert 10 pins. Performance was recorded with 2D motion capture (Vicon Motus) at 50 frames/s. Unsuccessful trials (i.e., trials with errors) were excluded from the analysis, on average 4% in the HC group and 5% in the MCI group. However, in case an error was made, an additional trial was recorded, to obtain 10 trials in each recording. In case of marker occlusion during performance, the resulting gaps were filled by cubic spline interpolation after recording. The coordinates of the reflective markers (Figure 2) were filtered with a low-pass Butterworth filter at the frequency of 10 Hz. After filtering, the recordings were segmented into four movement types: reaching, grasping, transport, and inserting of pins. The details of this segmentation have been previously described (Vasylenko et al., 2018): briefly, for reaching, onset was defined as the first frame of movement toward the cup and offset as the frame where the fingers were above the center of the cup; for grasping, onset was the first frame the fingers were lowered into the cup and offset the frame the pin was lifted out; for transport, onset was the first frame of movement toward the hole and offset the frame where the fingers reached the hole; for inserting, onset was the first frame the pin was lowered into the cup and the offset the frame the fingers were lifted off the pin. The reliability of this segmentation method ranged between 0.91 and 0.98, depending on movement type (Vasylenko et al., 2018). Kinematic measures were calculated separately for each movement type. Linear velocity, based on markers 1 and 4, represents the speed of movement for each hand. Path length, based on the same markers, represents the distance covered by each hand. Angular velocity, based on the angles 2-1-3 and 6-4-5, represents the speed of hand rotation. Angle, also based on markers 2-1-3 and 6-4-5, represents the position of the hand. In 2D recordings, larger angles indicate less pronated hand position. Coefficients of variation (CVs) of the kinematic parameters were also calculated. CV represents the extent of adjustments to a movement (e.g., corrective movements). All kinematic measures were calculated for each repetition of each movement and then averaged across the 10 repetitions of each movement type.

**Figure 2:**
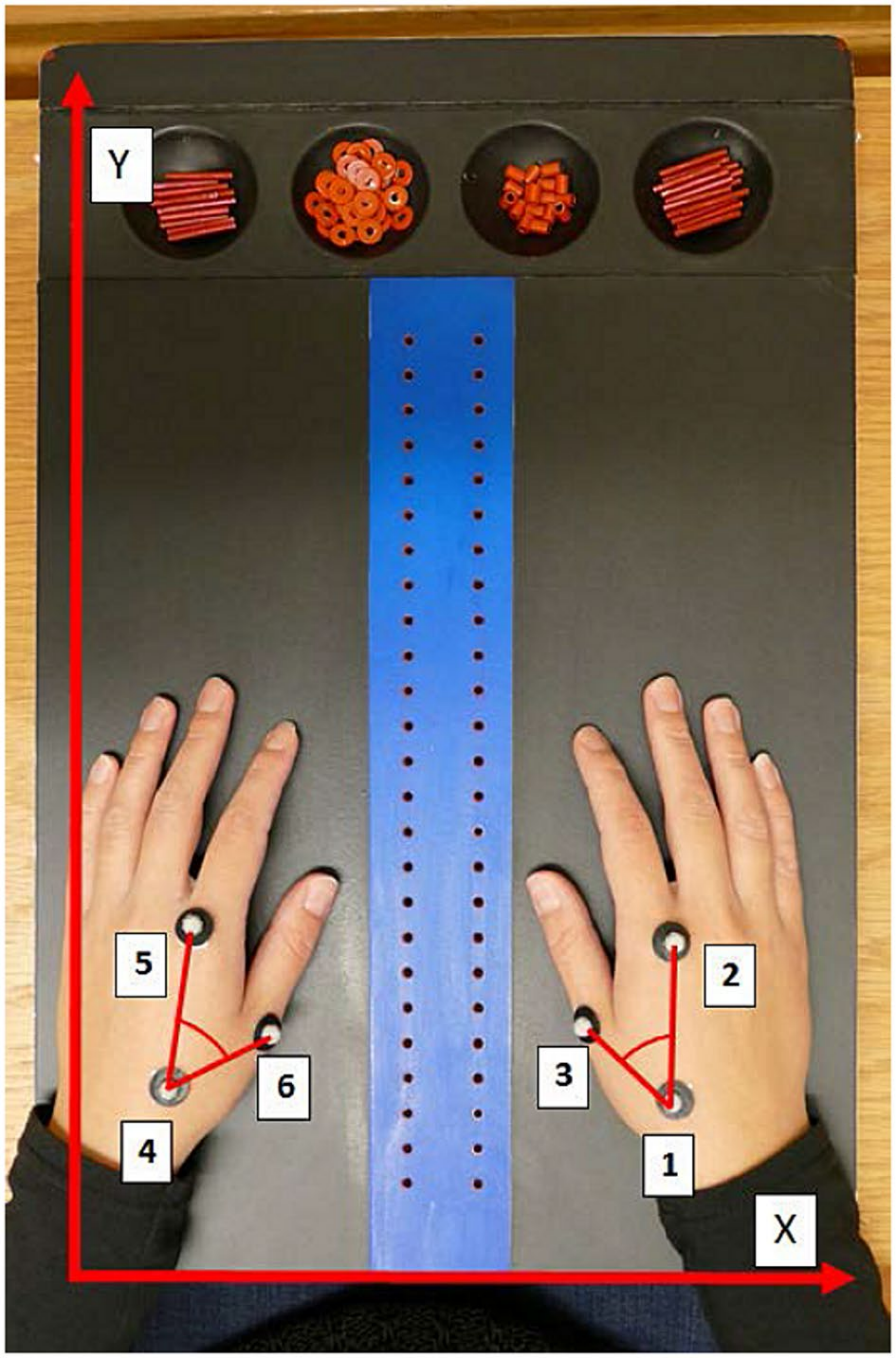
The Purdue Pegboard and marker arrangement, with axes and the angle used for analyses overlayed. Reproduced from Vasylenko et al. (2022) with permission from Wiley.

Laterality indices were calculated for all kinematic parameters and movement types with the formula (R − L)/(R + L), where R represents right hand- and L represents left hand performance. Ranging between −1 and +1, larger positive or negative values represent larger differences between right- and left-hand movements, while values closer to 0 represent more symmetric movements.

### 2.3 Procedure

Both testing sessions were conducted at the Department of Psychology, University of Tromsø. After administering the interview and screening measures, participants received a demonstration of the PPT tasks and were asked to practice until they were able to insert pins correctly. After practicing each task, they were instructed to perform as quickly as possible at the experimenter’s signal.

### 2.4 Statistical analyses

All participants had complete datasets across all tests and retest assessments. Group differences in demographics, handedness, and cognitive status were analyzed with independent samples *t*-tests. For hand dominance, the primary outcome measures were the seven LIs. Longitudinal changes in LIs were analyzed by repeated-measures MANOVAS with Time (T1, T2) as within-subjects factor and Group (HC, MCI) as between-subjects factor. The dependent variables (DVs) were the seven LIs. For manual dexterity, the primary outcome measures were the seven kinematic parameters. Longitudinal changes in kinematic parameters were analyzed by repeated-measures MANOVAs with Time (T1, T2) and Hand (right, left) as within-subjects factors, and Group (HC, MCI) as between-subjects factor. The DVs were the seven kinematic measures. In both analyses, separate MANOVAs were performed for each movement type. Due to the imbalanced gender ratio in both participant groups, all analyses were repeated with Gender as a covariate. This approach showed identical findings to the original analysis. Due to the difference in follow-up duration between the groups, all analyses were also repeated with Time interval between T1 and T2 as a covariate. The results of this analysis showed that Time interval somewhat reduced the multivariate effects of Time and Group, however, these effects, as well as Time × Group interactions, remained significant, while Time interval itself did not have significant multivariate effects on LIs or kinematic measures (see Supplementary Table 1 vs. Supplementary Table 2 for comparison). Pairwise comparisons of this approach showed almost identical findings to the original analyses (compare Supplementary Table 3 vs. Supplementary Table 4 for a representative example). Therefore, we report the findings of the original analyses. Significant omnibus effects were followed up by univariate ANOVAs. Greenhouse–Geisser correction was used in cases where the sphericity assumption was not met. Significant main effects and interactions were followed up by pairwise comparisons with Bonferroni correction. α was set at 0.05 for all tests. All analyses were performed with IBM SPSS Statistics version 29.

## 3 Results

The Results section is divided into three main areas presenting (a) demographic and background variables including cognitive status (section 3.1); (b) longitudinal results of the laterality indices (section 3.2) and (c) longitudinal results across all kinematic parameters (section 3.3). The last two sections comprise an additional sub-level (e.g., 3.2.1 Reaching) to present the findings for each of the four analyzed movements: Reaching, Grasping, Transport and Insert.

### 3.1 Demographics, handedness, and cognitive status

The groups did not differ in age, M (SD)_HC_ = 69.9 (3.92), M (SD)_MCI_ = 69.3 (6.60) at T1, M (SD)_HC_ = 73.5 (3.89), M (SD)_MCI_ = 74.6 (6.37) at T2, education, M (SD)_HC_ = 12.9 (2.63), M (SD)_MCI_ = 12.4 (4.33) or handedness, M (SD)_HC_ = 19.40 (5.24), M (SD)_MCI_ = 17.87 (11.15) at T1, M (SD)_HC_ = 19.55 (6.13), M (SD)_MCI_ = 19.07 (10.92) at T2. The MCI group had lower scores on MMSE at T1, M (SD)_HC_ = 29.35 (1.04), M (SD)_MCI_ = 26.50 (2.71), *t* (34) = 3.98, *p* < 0.001, 95% CI [1.35; 4.35]. At T2 the group difference in MMSE did not reach significance, M (SD)_HC_ = 28.15 (1.69), M (SD)_MCI_ = 25.63 (5.08), *t* (34) = 1.90, *p* = 0.073, 95% CI [−0.27; 5.32].

### 3.2 Longitudinal changes in laterality indices

The ANOVAs revealed significant main effects of Time, Group, and Time × Group interactions for all four movement types. Control analyses adjusting for the time interval between assessments (Δ *t*) as a covariate did not change the significance findings reported below. To emphasize longitudinal changes, we report significant effects that involve Time in the text. Effects of all factors are summarized in Supplementary Table 1 (multivariate effects) and Supplementary Table 5 (univariate effects). This applies to both LI and kinematic results.

#### 3.2.1 Reaching

There were significant Time × Group interactions for LI of linear velocity, *F*(1, 34) = 32.41, *p* < 0.001, *η*^2^*_p_* = 0.488, CV of linear velocity, *F*(1, 34) = 14.48, *p* < 0.001, *η*^2^*_p_* = 0.299, path length, *F*(1, 34) = 5.05, *p* = 0.031, *η*^2^*_p_* = 0.129, angle, *F*(1, 34) = 5.47, *p* = 0.025, *η*^2^*_p_* = 0.138, and CV of angle, *F*(1, 34) = 9.94, *p* = 0.003, *η*^2^*_p_* = 0.226. Pairwise comparisons of these interactions are summarized in Supplementary Table 3 and illustrated in Figure 3 In the HC group all the aforementioned LIs showed longitudinal decreases in right–left differences in performance, while no changes were found for the MCI group.

**Figure 3:**
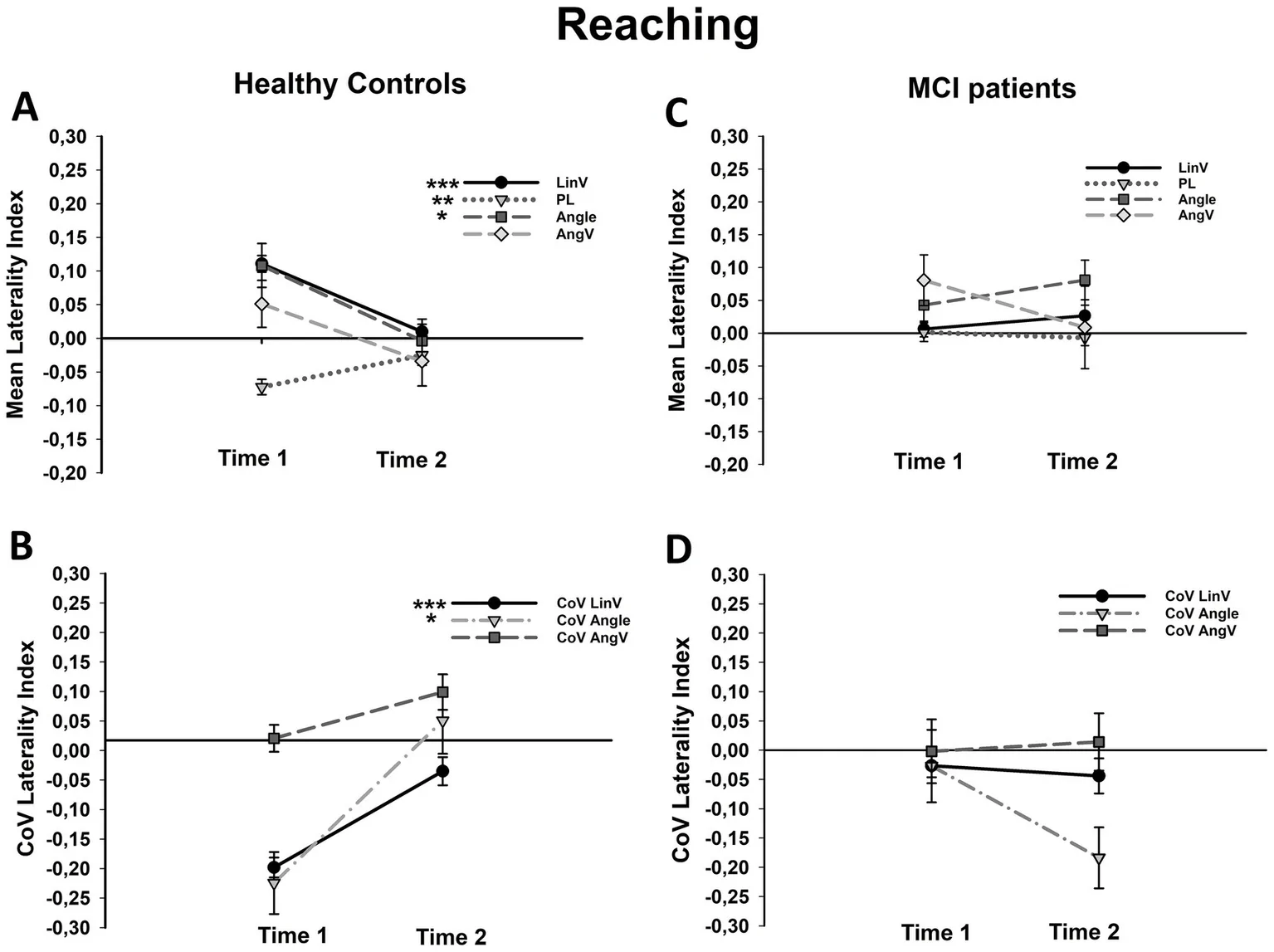
Mean ± SE laterality indices for kinematic variables during Reaching at first (Time 1) and second evaluation (Time 2). MCI, mild cognitive impairment; LinV, linear velocity (cm/s); PL, path length (cm); AngV, angular velocity (°/s). Mean values are portrayed on the top panels **(A,C)** and their respective coefficients of variation (CoV) are portrayed on the bottom panels **(B,D)**. * = *p* < 0.05; ** = *p* < 0.01; *** = *p* < 0.001.

#### 3.2.2 Grasping

For LI of linear velocity there were significant main effects of both Time, *F*(1, 34) = 5.57, *p* = 0.024, *η*^2^*_p_* = 0.141 and Group, *F*(1, 34) = 4.58, *p* = 0.040, *η*^2^*_p_* = 0.119. Significant Time × Group interactions were found for CV of linear velocity, *F*(1, 34) = 9.51, *p* = 0.004, *η*^2^*_p_* = 0.219, path length, *F*(1, 34) = 6.85, *p* = 0.013, *η*^2^*_p_* = 0.168, and angle, *F*(1, 34) = 6.88, *p* = 0.013, *η*^2^*_p_* = 0.168. Pairwise comparisons (Figure 3) revealed the same pattern of longitudinal changes as in reaching, with all the aforementioned LIs showing a decrease in right–left differences from T1 to T2 in the HC group but no changes in the MCI group (see Figure 4).

**Figure 4:**
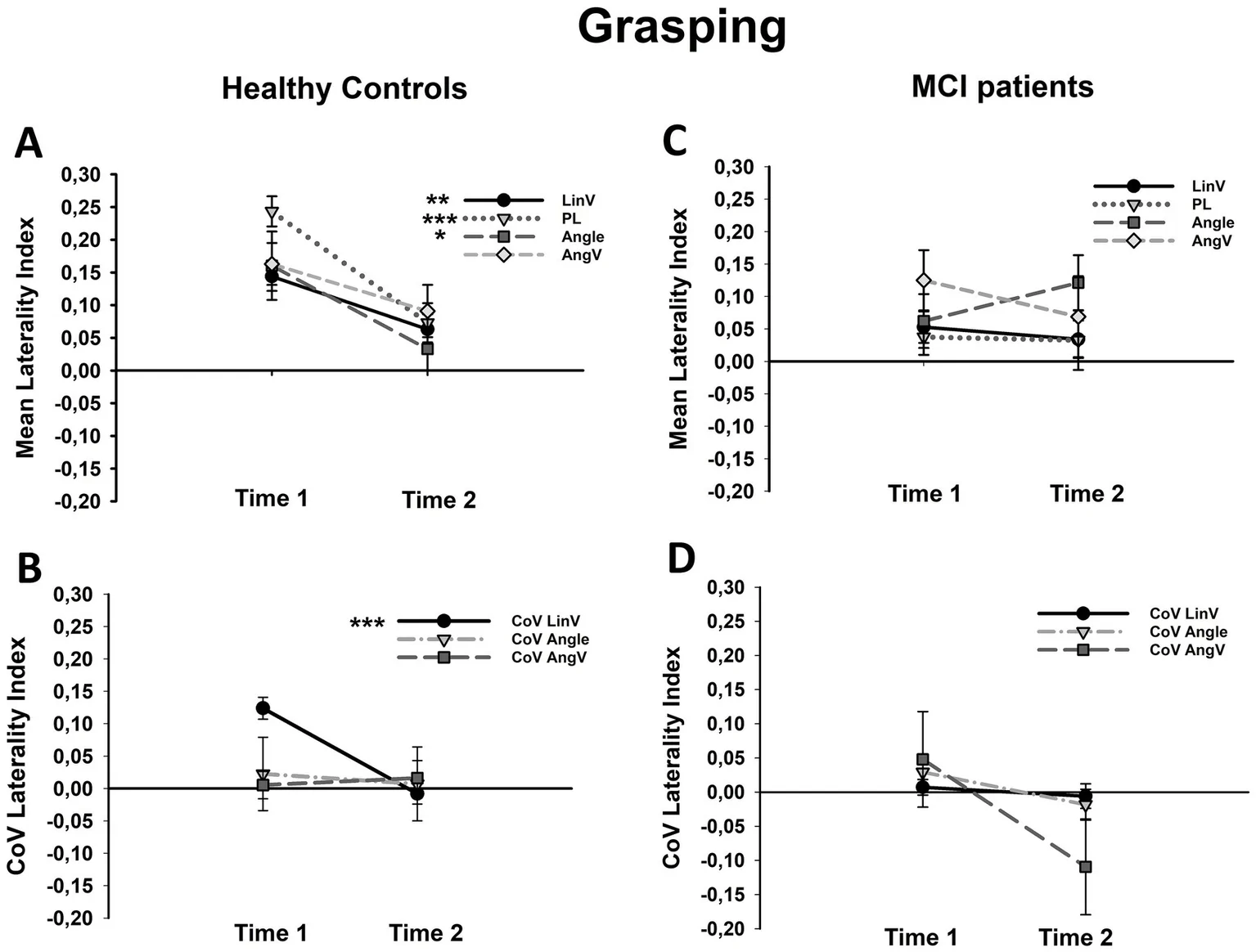
Mean ± SE laterality indices for kinematic variables during Grasping at first (Time 1) and second evaluation (Time 2). MCI, mild cognitive impairment. LinV, linear velocity (cm/s). PL, path length (cm). AngV, angular velocity (°/s). Mean values are portrayed on the top panels **(A,C)** and their respective coefficients of variation (CoV) are portrayed on the bottom panels **(B,D)**. * = *p* < 0.05; ** = *p* < 0.01; *** = *p* < 0.001.

#### 3.2.3 Transport

There were significant Time × Group interactions for linear velocity, *F*(1, 34) = 13.51, *p* < 0.001, *η*^2^*_p_* = 0.284, CV of linear velocity, *F*(1, 34) = 8.01, *p* = 0.008, *η*^2^*_p_* = 0.191, angular velocity, *F*(1, 34) = 5.70, *p* = 0.023, *η*^2^*_p_* = 0.144, CV of angular velocity, *F*(1, 34) = 6.86, *p* = 0.013, *η*^2^*_p_* = 0.168, and CV of angle, *F*(1, 34) = 8.43, *p* = 0.006, *η*^2^*_p_* = 0.199. Additionally, for path length there was a significant main effect of Time, *F*(1, 34) = 4.85, *p* = 0.035, *η*^2^*_p_* = 0.125. Pairwise comparisons of these effects are summarized in Figure 4. The HC group showed longitudinal decreases in right–left differences for all aforementioned LIs except angular velocity. The MCI group, on the other hand, showed a decrease in right–left differences for LI of angular velocity only (see Figure 5).

**Figure 5:**
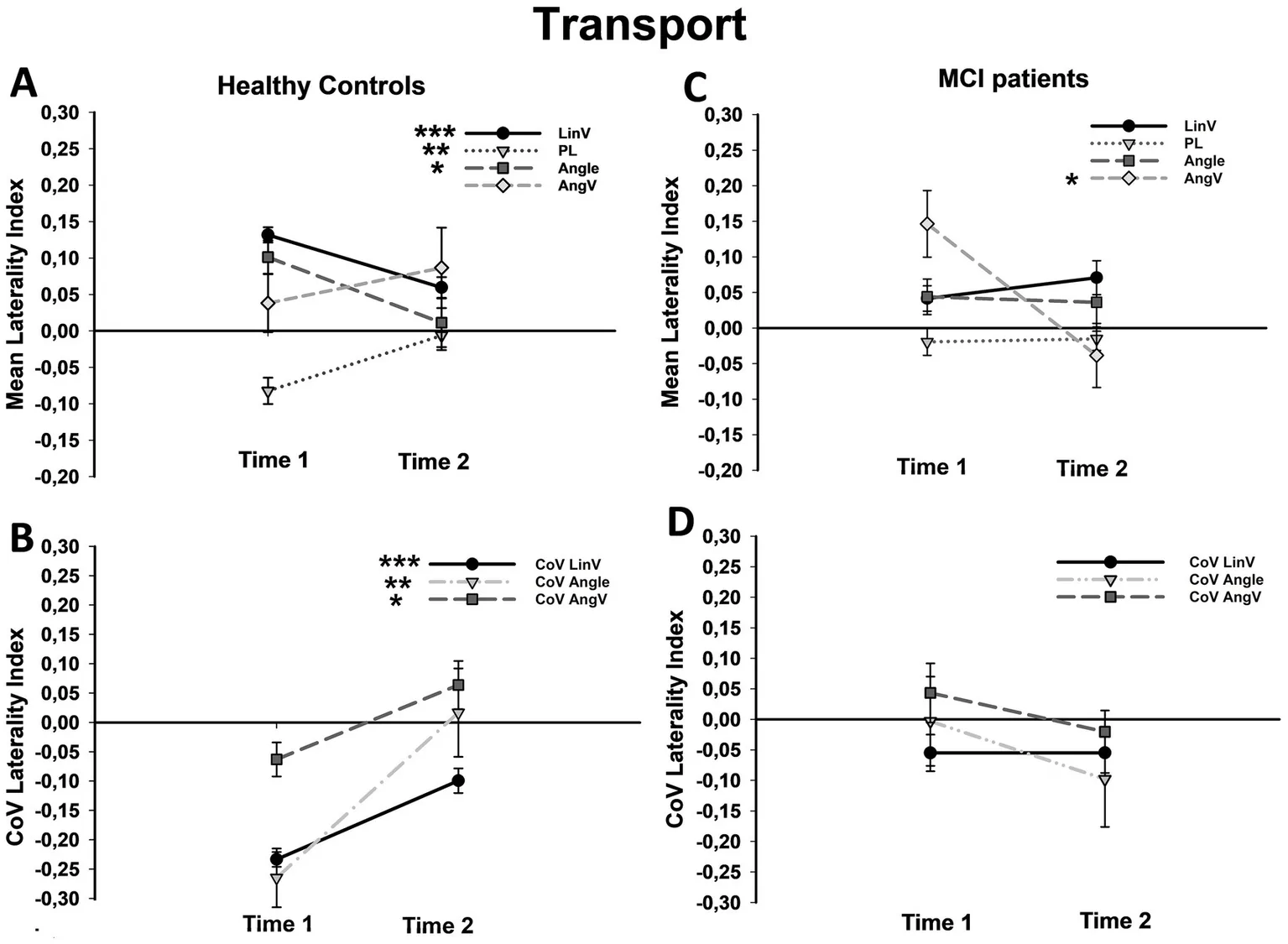
Mean ± SE laterality indices for kinematic variables during Transport at first (Time 1) and second evaluation (Time 2). MCI, mild cognitive impairment; LinV, linear velocity (cm/s); PL, path length (cm); AngV, angular velocity (°/s). Mean values are portrayed on the top panels **(A,C)** and their respective coefficients of variation (CoV) are portrayed on the bottom panels **(B,D)**. ^*^p < 0.05; ^**^p < 0.01; ^***^p < 0.001.

#### 3.2.4 Insert

There were significant Time × Group interactions for linear velocity, *F*(1, 34) = 5.65, *p* = 0.023, *η*^2^*_p_* = 0.142, CV of linear velocity, *F*(1, 34) = 5.07, *p* = 0.031, *η*^2^*_p_* = 0.130, and path length, *F*(1, 34) = 11.15, *p* = 0.002, *η*^2^*_p_* = 0.247. Figure 6 illustrates pairwise comparisons of these interactions. All aforementioned LIs showed decreases in right–left differences from T1 to T2 in the HC group, while in the MCI group there were no changes.

**Figure 6:**
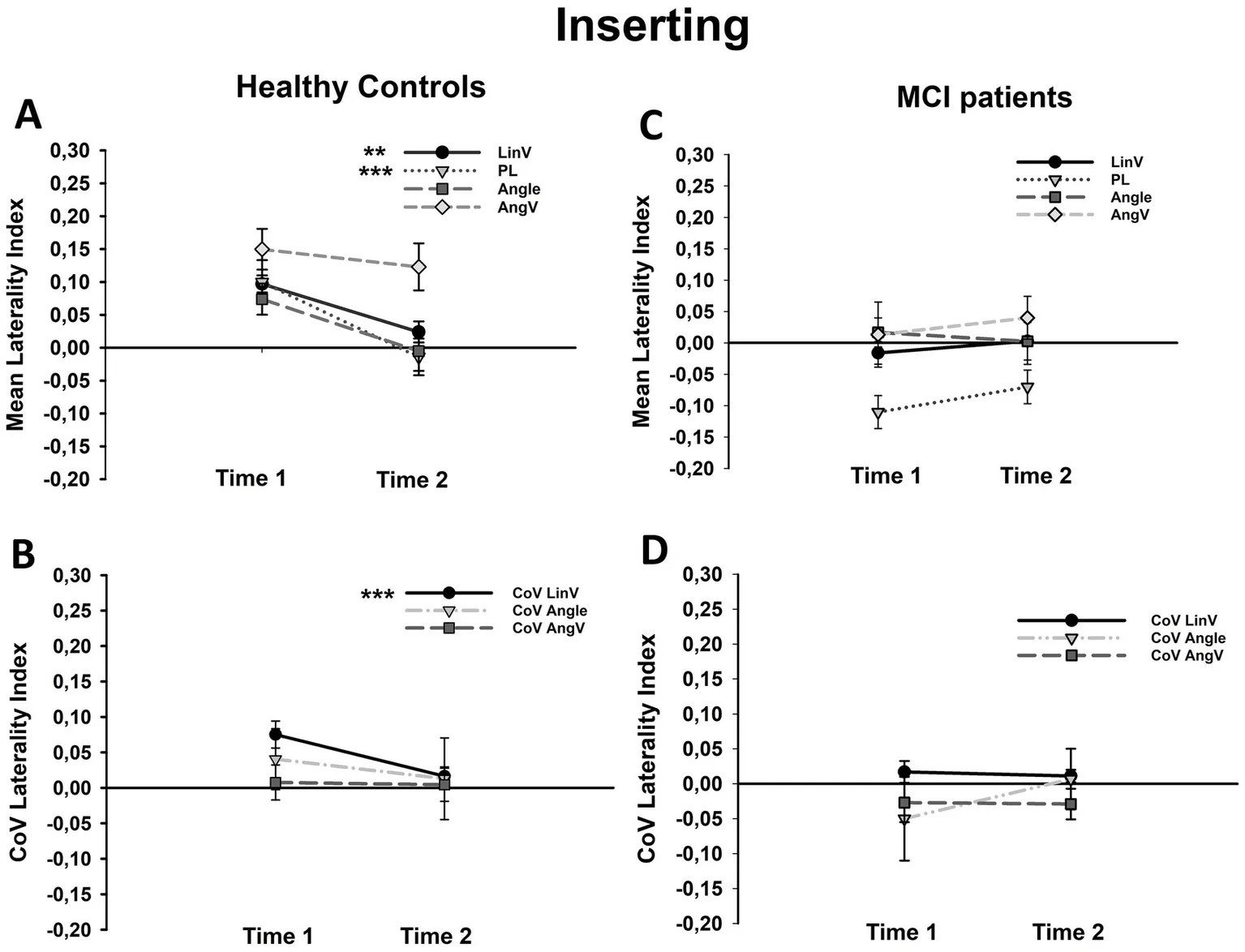
Mean ± SE laterality indices for kinematic variables during Inserting at first (Time 1) and second evaluation (Time 2). MCI, mild cognitive impairment; LinV, linear velocity (cm/s); PL, path length (cm); AngV, angular velocity (°/s). Mean values are portrayed on the top panels **(A,C)** and their respective coefficients of variation (CoV) are portrayed on the bottom panels **(B,D)**. ^**^p < 0.01; ^***^p < 0.001.

In summary, LI findings demonstrate longitudinal changes almost exclusively within the HC group, indicating more symmetric performance at T2 for all movement types in most of the kinematic parameters. These longitudinal shifts in the HC group are illustrated by individual LI trajectories for “*reaching”* and “*grasping*” actions (see Supplementary Figures 1, 2). In contrast, the MCI group showed virtually no longitudinal changes, thus preserving their symmetric performance from T1 to T2. To better appraise the shifts in the strength of lateralization between time points, the absolute LI values for both groups are provided in Supplementary Table 6.

### 3.3 Longitudinal changes in kinematic parameters

Means and SDs of all kinematic parameters are shown in Supplementary Table 7.

#### 3.3.1 Reaching

Time × Hand × Group interactions were significant for linear velocity, *F*(1, 34) = 25.13, *p* < 0.001, *η*^2^*_p_* = 0.425, CV of linear velocity, *F*(1, 34) = 26.28, *p* < 0.001, *η*^2^*_p_* = 0.436, path length, *F*(1, 34) = 7.38, *p* = 0.01, *η*^2^*_p_* = 0.178, angle, *F*(1, 34) 5.21, *p* = 0.029, *η*^2^*_p_* = 0.133, and CV of angle, *F*(1, 34) = 13.80, *p* < 0.001, *η*^2^*_p_* = 0.289. Pairwise comparisons of these interactions are summarized in Table 1. Specifically, there was an increase in linear velocity from T1 to T2 in the HC group, but only for the left hand. In the MCI group, linear velocity decreased over time in both hands. CV of linear velocity decreased in the HC group while it increased in the MCI group. Path length decreased in the HC group, while in the MCI group there were no changes. Angle showed changes in the left hand only, increasing in the HC group and decreasing in the MCI group over time. CV of angle also showed changes in the left hand only, decreasing in the HC group and increasing in the MCI patients.

**Table 1:** Pairwise comparisons of Time × Hand × Group interactions for reaching.

Kinematic	Right hand									
	HC					MCI				
	T1 [M (SD)]	T2 [M (SD)]	*t*	*p*	95% CI	T1 [M (SD)]	T2 [M (SD)]	*t*	*p*	95% CI
LinV	46.35 (7.76)	49.59 (8.38)	−1.53	0.135	[−7.54; 1.06]	**44.94 (7.76)**	**39.19 (8.38)**	**2.43**	**0.021**	**[0.94; 10.56]**
CV linV	**0.32 (0.05)**	**0.23 (0.04)**	**5.93**	**<0.001**	**[0.06; 0.12]**	**0.26 (0.05)**	**0.32 (0.05)**	**−3.53**	**<0.001**	**[−0.09; −0.03]**
PL	**138.93 (14.50)**	**117.80 (14.55)**	**5.28**	**<0.001**	[13.0; 29.25]	120.99 (14.50)	118.42 (14.56)	0.57	0.570	[−6.52; 11.65]
Angle	44.98 (10.77)	46.25 (11.92)	−0.42	0.675	[−7.41; 4.85]	47.92 (10.77)	43.88 (11.92)	1.20	0.239	[−2.81; 10.90]
CV angle	0.13 (0.07)	0.11 (0.08)	0.85	0.389	[−0.03; 0.06]	0.15 (0.07)	0.18 (0.08)	−1.25	0.217	[−0.08; 0.02]

Kinematic	Left hand									
	HC					MCI				
	T1 [M (SD)]	T2 [M (SD)]	*t*	*p*	95% CI	T1 [M (SD)]	T2 [M (SD)]	*t*	*p*	95% CI
LinV	**36.93 (7.03)**	**48.54 (7.74)**	**−6.51**	**<0.001**	**[−15.24; −7.99]**	**44.47 (7.03)**	**37.04 (7.74)**	**3.73**	**<0.001**	**[3.38; 11.49]**
CV linV	**0.48 (0.06)**	**0.25 (0.06)**	**12.32**	**<0.001**	**[0.20; 0.27]**	**0.27 (0.06)**	**0.35 (0.06)**	**−3.81**	**<0.001**	**[−0.12; −0.04]**
PL	**160.44 (16.85)**	**124.02 (17.66)**	**8.52**	**<0.001**	**[27.73; 45.09]**	121.13 (16.84)	120.90 (17.67)	0.05	0.962	[−9.48; 9.94]
Angle	**36.35 (10.55)**	**46.53 (9.63)**	**−3.40**	**0.002**	**[−16.25; −4.10]**	**44.22 (10.56)**	**36.87 (9.64)**	**2.20**	**0.035**	**[0.55; 14.14]**
CV angle	**0.20 (0.08)**	**0.11 (0.05)**	**4.95**	**<0.001**	**[0.06; 0.13]**	**0.16 (0.07)**	**0.25 (0.06)**	**−4.52**	**<0.001**	**[−0.14; −0.05]**

HC, healthy controls; MCI, mild cognitive impairment; T1, first test session; T2, retest session; LinV, linear velocity (cm/s); CV, coefficient of variability (SD/M); PL, path length (cm). M values represent model-estimated EM means. SD values represent model-estimated SEM × √*n*. Bold values represent statistically significant pairwise comparisons.

Additionally, significant Time × Group interactions were found for angular velocity, *F*(1, 34) = 10.74, *p* = 0.002, *η*^2^*_p_* = 0.240, and CV of angular velocity, *F*(1, 34) = 8.55, *p* = 0.006, *η*^2^*_p_* = 0.201. Pairwise comparisons revealed that these changes were significant for the HC group only. Specifically, there was a decrease over time in both angular velocity, M (SD)_T1_ = 68.34 (14.50), M (SD)_T2_ = 51.61 (14.22), *t*(34) = 4.0, *p* < 0.001, 95% CI [8.23; 25.21] and in CV of angular velocity, M (SD)_T1_ = 0.69 (0.11), M (SD)_T2_ = 0.55 (0.10), *t*(34) = 4.57, *p* < 0.001, 95% CI [0.08; 0.20].

#### 3.3.2 Grasping

Significant Time × Hand × Group interactions were found for CV of linear velocity, *F*(1, 34) = 6.88, *p* = 0.013, *η*^2^*_p_* = 0.168, and angle, *F*(1, 34) = 4.59, *p* = 0.039, *η*^2^*_p_* = 0.119. Pairwise comparisons of these interactions showed longitudinal changes only in the HC group and only for the left hand. Specifically, there were increases over time both in CV of linear velocity, M (SD)_T1_ = 0.52 (0.08), M (SD)_T2_ = 0.73 (0.09), *t*(34) = −8.83, *p* < 0.001, 95% CI [−0.25; −0.16], and in angle, M (SD)_T1_ = 31.71 (13.09), M (SD)_T2_ = 43.84 (15.11), *t*(34) = −3.43, *p* = 0.002, 95% CI [−19.32; −4.95].

Time × Group interactions were significant for linear velocity, *F*(1, 34) = 35.27, *p* < 0.001, *η*^2^*_p_* = 0.509, path length, *F*(1, 34) = 21.09, *p* < 0.001, *η*^2^*_p_* = 0.383, angular velocity, *F*(1, 34) = 6.99, *p* = 0.012, *η*^2^*_p_* = 0.171, and CV of angular velocity, *F*(1, 34) = 17.99, *p* < 0.001, *η*^2^*_p_* = 0.346. Pairwise comparisons of these interactions (see Table 2) showed longitudinal changes in both groups. In the HC group, there was an increase in linear velocity and path length, as well as a decrease in CV of angular velocity. The MCI group showed decreases in linear and angular velocities and an increase in CV of angular velocity.

**Table 2:** Pairwise comparisons of Time × Group interactions for grasping and inserting.

Kinematic	Grasping									
	HC					MCI				
	T1 [M (SD)]	T2 [M (SD)]	*t*	*p*	95% CI	T1 [M (SD)]	T2 [M (SD)]	*t*	*p*	95% CI
LinV	**7.47 (1.44)**	**10.24 (1.67)**	**−5.57**	**<0.001**	**[−3.78; −1.76]**	**8.64 (1.44)**	**6.98 (1.67)**	**2.98**	**0.005**	**[0.54; 2.78]**
PL	**64.62 (19.41)**	**91.42 (19.19)**	**−5.27**	**<0.001**	**[−37.12; −16.47]**	81.25(19.42)	73.05(19.19)	1.44	0.158	[−3.35; 19.74]
AngV	48.68 (13.02)	53.82 (15.96)	−1.33	0.191	[−12.96; 2.68]	**59.66 (13.03)**	**49.54 (15.98)**	**2.35**	**0.025**	**[1.38; 18.87]**
CV angV	**0.75 (0.07)**	**0.67 (0.10)**	**3.59<**	**0.001**	**[0.04; 0.12]**	**0.66 (0.07)**	**0.72 (0.10)**	**−2.46**	**0.020**	**[−0.11; −0.01]**

Kinematic	Inserting									
	HC					MCI				
	T1 [M (SD)]	T2 [M (SD)]	*t*	*p*	95% CI	T1 [M (SD)]	T2 [M (SD)]	*t*	*p*	95% CI
CV linV	**0.67 (0.06)**	**0.79 (0.07)**	**−7.35**	**<0.001**	**[−0.16; −0.09]**	0.76(0.06)	0.73 (0.07)	1.79	0.082	[−0.004; 0.07]
CV angV	**0.91 (0.13)**	**0.79 (0.11)**	**4.52**	**<0.001**	**[0.07; 0.18]**	0.69(0.12)	0.69 (0.11)	0.03	0.985	[−0.06; 0.06]
Angle	**45.21 (10.0)**	**52.37 (8.88)**	**−2.86**	**0.007**	**[−12.25; −2.07]**	**56.29(10.0)**	**46.65 (8.88)**	**3.45**	**0.002**	**[3.96; 15.33]**

HC, healthy controls; MCI, mild cognitive impairment; T1, first test session; T2, retest session; LinV, linear velocity (cm/s); PL, path length (cm); AngV, angular velocity (°/s); CV, coefficient of variability (SD/M). M values represent model-estimated EM means. SD values represent model-estimated SEM × √*n*. Bold values represent statistically significant pairwise comparisons.

#### 3.3.3 Transport

Time × Hand × Group interactions were significant for linear velocity, *F*(1, 34) = 7.76, *p* = 0.009, *η*^2^*_p_* = 0.186, CV of linear velocity, *F*(1, 34) = 19.96, *p* < 0.001, *η*^2^*_p_* = 0.370, path length, *F*(1, 34) = 5.17, *p* = 0.029, *η*^2^*_p_* = 0.132, angular velocity, *F*(1, 34) = 4.94, *p* = 0.033, *η*^2^*_p_* = 0.127, CV of angular velocity, *F*(1, 34) = 8.23, *p* = 0.007, *η*^2^*_p_* = 0.195, and CV of angle, *F*(1, 34) = 5.01, *p* = 0.032, *η*^2^*_p_* = 0.128. Results of pairwise comparisons for these interactions are shown in Table 3. Linear velocity increased from T1 to T2 in the HC group, in the left hand only, while it decreased in both hands in the MCI group. CV of linear velocity, path length, and CV of angular velocity showed changes only for the HC group, with all three parameters decreasing in both hands over time. Angular velocity also decreased in both hands in the HC group, while in the MCI group the decrease was significant only for the right hand. CV of angle showed changes in left-hand movements for both groups, decreasing in the HC group and increasing in the MCI group.

**Table 3:** Pairwise comparisons of Time × Hand × Group interactions for transport.

Kinematic	Right hand									
	HC					MCI				
	T1 [M (SD)]	T2 [M (SD)]	*t*	*p*	95% CI	T1 [M (SD)]	T2 [M (SD)]	*t*	*p*	95% CI
LinV	35.67 (4.79)	36.40 (6.48)	−0.49	0.627	[−3.75; 2.29]	**32.25 (4.79)**	**28.37 (6.49)**	**2.34**	**0.025**	**[0.51; 7.25]**
CV linV	0**.28 (0.04)**	**0.23 (0.04)**	**3.62**	**<0.001**	**[0.02; 0.67]**	0.27 (0.04)	0.29 (0.04)	−1.79	0.089	[−0.05; 0.004]
PL	**108.26 (14.15)**	**92.25 (11.18)**	**4.25**	**<0.001**	**[8.36; 23.67]**	91.41 (14.16)	88.22 (11.18)	0.76	0.455	[−5.38; 11.75]
AngV	**62.44 (21.72)**	**47.86 (16.25)**	**2.48**	**0.018**	**[2.62; 26.54]**	**73.60 (21.74)**	**47.28 (16.26)**	**4.0**	**<0.001**	**[12.95; 39.69]**
CV angV	**0.73 (0.13)**	**0.65 (0.12)**	**2.0**	**0.049**	**[0.001; 0.16]**	0.51 (0.13)	0.50 (0.12)	0.17	0.849	[−0.08; 0.10]
CV angle	0.09(0.05)	0.07 (0.08)	1.17	0.252	[−0.02; 0.06]	0.11 (0.05)	0.14 (0.08)	−1.25	0.234	[−0.07; 0.02]

Kinematic	Left hand									
	HC					MCI				
	T1 [M (SD)]	T2 [M (SD)]	*t*	*p*	95% CI	T1 [M (SD)]	T2 [M (SD)]	*t*	*p*	95% CI
LinV	**27.44 (4.83)**	**32.15(5.94)**	**−4.1**	**<0.001**	**[−7.05; −2.38]**	**29.78(4.83)**	**25.06 (5.94)**	**3.68**	**<0.001**	**[2.11; 2.34]**
CV linV	0.**45 (0.05)**	**0.28 (0.06)**	**3.32**	**<0.001**	**[0.14; 0.20]**	0.30 (0.06)	0.33 (0.06)	−2.0	0.057	[−0.001; 0.02]
PL	**127.94 (17.29)**	**93.92 (14.30)**	**7.36**	**<0.001**	**[24.63; 43.41]**	94.97 (17.30)	91.06 (14.32)	0.76	0.455	[−6.59; 14.41]
AngV	**56.55 (18.54)**	**43.61 (21.36)**	**2.54**	**0.016**	**[2.62; 23.25]**	54.23 (18.55)	52.94 (21.37)	0.23	0.821	[−10.24; 12.82]
CV angV	**0.82 (0.12)**	**0.58 (0.13)**	**6.67**	**<0.001**	**[1.68; 0.31]**	0.47 (0.12)	0.52 (0.13)	−1.23	0.232	[−0.13; 0.03]
CV angle	**0.16 (0.07)**	**0.07 (0.06)**	**5.73**	**<0.001**	**[0.06; 0.12]**	**0.11 (0.06)**	**0.15 (0.06)**	**−2.06**	**0.041**	**[−0.07; −0.002]**

HC, healthy controls; MCI, mild cognitive impairment; T1, first test session; T2, retest session; LinV, linear velocity (cm/s); CV, coefficient of variability (SD/M); PL, path length (cm); AngV, angular velocity (°/s). M values represent model-estimated EM means. SD values represent model-estimated SEM × √*n*. Bold values represent statistically significant pairwise comparisons.

For angle, there was a significant Time × Group interaction, *F*(1, 34) = 14.32, *p* < 0.001, *η*^2^*_p_* = 0.296, showing larger angles at T2 in the HC group, M(SD)_T1_ = 52.94(8.58), M(SD)_T2_ = 60.13(8.46), t(34) = −2.84, *p* = 0.007, 95% CI [−12.32; −2.05], but smaller angles in the MCI group, M(SD)_T1_ = 60.81(8.58), M(SD)_T2_ = 53.66(8.46), t(34) = 2.53, *p* = 0.016, 95% CI [1.41; 12.89].

#### 3.3.4 Inserting

Significant Time × Hand × Group interactions were found for linear velocity, *F*(1, 34) = 5.15, *p* = 0.030, *η*^2^*_p_* = 0.132, and path length, *F*(1, 34) = 9.84, *p* = 0.004, *η*^2^*_p_* = 0.225. Pairwise comparisons showed significant changes for the HC group only, with increases in the left hand both for linear velocity, M (SD)_T1_ = 6.47 (1.45), M (SD)_T2_ = 7.68 (1.65), *t*(34) = −3.0, *p* = 0.005, 95% CI [−2.03; −0.39], and path length, M (SD)_T1_ = 60.47 (20.90), M (SD)_T2_ = 75.42 (18.97), *t*(34) = −3.11, *p* = 0.004, 95% CI [−24.71; −5.20].

Time × Group interactions were significant for CV of linear velocity, *F*(1, 34) = 39.76, *p* < 0.001, *η*^2^*_p_* = 0.539, CV of angular velocity, *F*(1, 34) = 8.76, *p* = 0.006, *η*^2^*_p_* = 0.205, and angle, *F*(1, 34) = 20.02, *p* < 0.001, *η*^2^*_p_* = 0.371. Pairwise comparisons of these interactions are shown in the lower part of Table 2. In the HC group, there were significant longitudinal changes for all three parameters, showing an increase in CV of linear velocity, a decrease in CV of angular velocity, and an increase in angle. In the MCI group, the only significant change was for angles, which decreased over time.

In summary, longitudinal changes in kinematic parameters suggest improved gross dexterity (reaching and transport movements) in healthy controls, as indicated by an increase in linear velocity, decrease in path length and movement variability. In contrast, for the MCI patients, the findings suggest declines in gross dexterity, as indicated by a decrease in linear velocity and increase in variability. For fine dexterity (grasping and inserting movements) of healthy controls, longitudinal findings also suggest improvements, as indicated by increases in linear velocity and decreases in CV of angular velocity, but also declines, as indicated by increases in CV of linear velocity and path length. For MCI patients, longitudinal findings suggest decline in grasping movements, as indicated by decreases in linear and angular velocities and increases in CV of angular velocity. However, very limited changes were found in inserting movements for this group, suggesting mostly preserved performance over time. When comparing longitudinal changes between the dominant and non-dominant hands across all movement types, the findings suggest that in the HC group kinematic changes were more extensive in the non-dominant hand, while in the MCI group they were more uniform for both hands. To facilitate appraisal of these findings we have illustrated the longitudinal gains and losses in kinematic measures of two representative movements for gross (reaching) and fine (grasping) motor function through colored tables by group and hand (see Figure 7). In this figure, blue colors indicate better performance, while red colors denote worsened performance.

**Figure 7:**
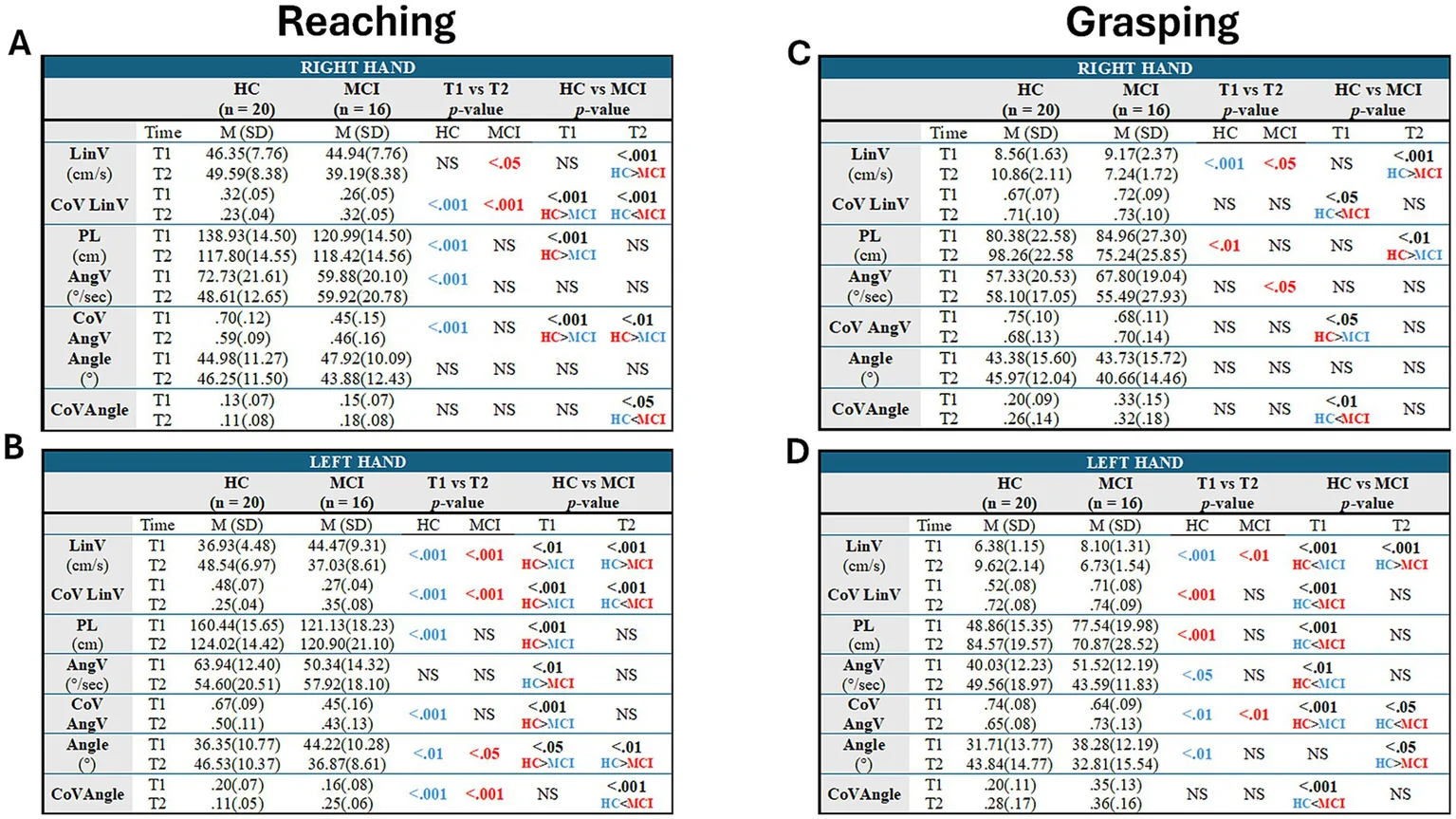
Illustration of longitudinal gains (blue) and losses (red) on reaching and grasping. Upper tables **(A,C)** show data from the right hand, while lower tables show data for the left hand **(B,D)**. The tables present mean values (M), standard deviations (SD) and *p*-values of pairwise comparisons for each kinematic parameter. HC, healthy controls; MCI, mild cognitive impairment; T1, assessment at Time 1; T2, assessment at Time 2; LinV, linear velocity (cm/s); CV, coefficient of variability (SD/M); PL, path length (cm); AngV, angular velocity (°/s).

## 4 Discussion

The present investigation demonstrates that right-handed healthy older adults lose their asymmetric hand dominance after 3.5 years of initial testing. In addition, we confirmed that MCI patients preserved symmetric hand function after 5.4 years of first evaluation. These findings suggest that independently of their group affiliation (i.e., patients vs. controls), all tested individuals displayed symmetric hand dominance after a longitudinal examination at different points in time. Our previous study reporting cross-sectional results of the first evaluation (Vasylenko et al., 2022) showed that laterality indices of kinematic parameters differentiated patients from controls. For this reason, we proposed that the assessment of hand dominance in early stages of cognitive decline should be used for the identification of MCI individuals.

Now, the longitudinal results suggest that the asymmetric hand dominance established earlier in life evolves into more symmetric performance in late adulthood regardless of whether individuals suffer or not from cognitive deterioration. Therefore, the development of symmetric dexterity should be regarded as inherent to the aging process and not as a unique disease-specific marker of MCI. The clear group difference in LIs at the initial evaluation should be attributed to accelerated brain aging in patients, which is thought to be a central factor for the manifestation of cognitive deterioration (Huang et al., 2021). In fact, various studies have demonstrated that the MCI condition exacerbates age-related changes in the brain, cognitive and motor functions (Buracchio et al., 2010; Ly et al., 2024). Thus, our data aligns with these observations and the suggestion that MCI accelerates the appearance of symmetric hand dexterity.

### 4.1 Understanding symmetric hand dominance in MCI patients and controls

The finding of comparable LIs in our groups brings the question of how to interpret the longitudinal results of laterality indices on the tested samples. To address this issue, a close inspection of the kinematic parameters is deemed necessary. Linear and angular measurements were extracted for each hand during four predefined movements including two actions for fine dexterity (i.e., grasping and inserting) and two for gross dexterity (i.e., reaching and transport). MCI patients in general demonstrated less pronounced changes from test to re-test evaluations across the kinematic outcomes. However, significant changes occurred during both gross dexterity and grasping, showing deterioration of hand function. Interestingly, the ability to insert was almost unchanged, with only significant declines in mean angles. Overall, the above findings for MCI patients indicate a worsening in dexterity of both hands, with a more prominent decline in the non-dominant hand. Similar findings of reduced ability of the non-dominant hand have been reported in cross-sectional studies (Suzumura et al., 2018; Suzumura et al., 2016). One plausible explanation of the more pronounced left-hand deterioration in MCI is a greater atrophy on the right brain hemisphere reported in some studies (Apostolova et al., 2007; Bell-McGinty et al., 2005; Zhang et al., 2021).

As for the healthy older group, important changes in dexterity were found at the re-test assessment. On one side, the longitudinal data showed age-related declines well documented in the literature related to fine dexterity during grasping and inserting (Diermayr et al., 2011; Krampe, 2002; Voelcker-Rehage and Alberts, 2005). On the other side we found changes in kinematic parameters denoting improvement of gross dexterity. Especially, more dexterous execution of the non-dominant hand was observed at re-test, which agrees with previous cross-sectional studies reporting better performance of the non-dominant hand of healthy older adults (Pan et al., 2023; Przybyla et al., 2011). The improvement of dexterity was more notable during reaching and transport, but enhanced performance was also found for some kinematic measures during fine movements, i.e., grasping and inserting.

As a whole, improved performance in the healthy group indicates, to some degree learning effects in both hands, albeit at different rates. In agreement with the literature (Pan et al., 2023; Turner et al., 2011), we hypothesize that the long lifetime use of the dominant hand promotes not only dexterous ability but also excessive use of it, which causes strenuous movements and higher vulnerability to damage to the right hand. Thus, this overuse hypothesis may help explain why improvements in the dominant hand are less pronounced than those of the non-dominant hand at retest. Although no causal evidence exists, we speculate that the right hand reaches a ceiling capacity for compensation. This may be driven by a combination of normal neurophysiological losses, such as declines in hand strength, muscle mass, and deterioration of motor neuron properties (Tudorascu et al., 2014) combined with selective excessive damage from overuse.

As for the improvements of the non-dominant hand, our explanation is that an interlimb transfer of learning (Stockinger et al., 2015) is taking place, which is consistent with previous findings demonstrating symmetric interlimb transfer in visuomotor adaptation among older participants (Wang et al., 2011). Even though we lack mechanistic evidence for this proposal, we put forward the idea that as the dominant hand’s superiority becomes limited, new motor adaptations need to arise. In this scenario, the development of symmetric hand dexterity in late adulthood is compensatory and it is reasonable to believe that such a change in motor behavior entails changes in neural plasticity that might contribute to the age-related reorganization of brain function (Gooijers et al., 2024; Zatorre et al., 2012). Accordingly, it seems plausible that motor adaptations like the one presented in our study drive, to some extent, the hemispheric reorganization described in the HAROLD model. Nevertheless, since we lack empirical support for this explanation, other alternative reasons should also be considered, including regression to the mean, test–retest effects, changes in measurement procedures, and the fixed order of the tasks in our study (i.e., the right hand always assessed before the left hand).

In the past, the reasons for the emergence of symmetric hand dexterity in older adults have been attributed to tentative factors such as reduced hemispheric cerebral activity, lifestyle and environmental changes (Kalisch et al., 2006; Sebastjan et al., 2017). However, our longitudinal data showing clear gains and improvements in dexterity in the control group suggest that a preserved ability for motor learning in healthy individuals might be a central process behind the emergence of symmetric hand dominance. The fact that improved performance at retest is better for the non-dominant hand suggests a more intact neuromuscular system of the left hand. Since we did not have any measure for the integrity of the neuromuscular system of the upper limbs, this suggestion needs to be followed up in future research. For instance, it would be important to measure the integrity and extent to which the neuromuscular junction has been remodeled on each upper limb. It is acknowledged that a loss of motor neurons occurs in aging (Campbell et al., 1973), which leads to a certain degree of permanent loss but also to the remodeling of surviving motor units (Tudorascu et al., 2014). It is estimated that healthy older adults over the age of 70 have around 40% fewer motor units than early in life, and these changes are accompanied by neuromuscular remodeling, which has important implications for the control of movement (Piasecki et al., 2016).

Based on the aforementioned hypotheses, we suggest that healthy older adults develop more symmetric performance due to the emergence of new behavioral patterns that compensate for limitations of the dominant hand. Although direct empirical support is currently lacking, our findings provide a strong rationale suggesting that this phenomenon reflects an increased capacity for interlimb learning in the non-dominant hand. This capacity, in turn, may be related to better neurophysiological integrity and preserved cognitive functioning. In contrast, MCI patients exhibited limited compensation, maintaining their symmetric hand dexterity 4.5 years after the initial assessment. Although the MCI group showed no change in various kinematic parameters at retest, they demonstrated an evident worsening in hand dexterity, which most likely relates to their progressive cognitive deterioration.

### 4.2 Limitations and future venues

The present study has some limitations. To begin with, the study operated with a relatively small sample in both groups. Also, the selection of the MCI group was restricted to those individuals who had a certain degree of preserved capabilities that allowed them to participate in the longitudinal evaluation. As such, we ended up with a group of MCI patients that were relatively stable after a long period, which limits generalizability. Also, the meantime interval between test and retest in both groups differed by more than 2 years, which is an important source of variation. Still, despite the differences in time intervals for retesting, the phenomenon of reduced manual asymmetry is notable in both groups. Further issues that might affect the observed findings relate to the type of instruction given. The command to perform “as quickly as possible” may adversely affect performance in complex motor tasks compared to the ‘preferred pace’ instruction, especially in older adults with neurodegenerative disorders (Shaw et al., 2011). Therefore, the question of how the type of instruction affects changes in hand dominance is an important one and should be examined in future research. In addition, other factors not addressed in this study such as age-related cerebral changes should not be underestimated. Indeed, brain degeneration due to normal aging or to age-related pathological conditions like MCI might be influencing the findings reported in the present study and the neural correlates of reduced manual asymmetry in older age need to be clarified in future research.

A final limitation involves the absence of a detailed annualized analysis regarding shifts in hand preference. Nonetheless, since the present study represents a foundational first step toward understanding the complex emergence of symmetric hand dominance in old age, establishing these initial parameters was necessary before annual trajectories could be precisely tracked. While a detailed annualized analysis fell outside the scope of this current work, future research should leverage longitudinal designs to track these dynamic changes over time.

Lastly, a methodological consideration needs to be made. Since this study adopted a 2D analysis, a pertinent question is whether dexterity performance would vary under a 3D framework, for instance, if distinct effects emerge across different dimensions. To answer the question, a comparison of the results from 2D vs. 3D approaches need to be conducted. However, to our knowledge no one has reported such investigation in the literature. Still, based on the nature of movements analyzed and the properties of 2D and 3D motion capture, it is possible to make some predictions. First, gross movements (reaching and transport) mostly occur along the XY plane, whereas fine movements (grasping and inserting) have more movement along the Z axis. Therefore, we would not expect large differences between 2D and 3D results for the gross movements, however, for the fine movements we predict longer path lengths as the 3D analysis would capture the additional Z component not captured by the 2D analysis. Secondly, because 3D capture involves several cameras while 2D capture is limited to one, we would expect fewer marker occlusions and more precise data with 3D analysis, likely resulting in less movement variability in the results. Overall, we estimate that the primary benefit of a 3D approach will be the acquisition of highly precise data on fine hand dexterity, rather than any fundamental shifts in our findings. Securing such refined metrics in the future is a highly pertinent issue that would greatly enrich the present investigation.

## 5 Conclusion

To conclude, we wish to emphasize that the phenomenon of symmetric hand function in older adults is not new in the literature and various cross-sectional studies have reported it (Kalisch et al., 2006; Pan et al., 2023; Przybyla et al., 2011; Sebastjan et al., 2017). However, to the best of our knowledge, no previous data exist documenting the longitudinal development of symmetric hand dexterity in aging populations. Hence, the present study is the first one to demonstrate longitudinally the actual maintenance and progression into more symmetric hand performance across two groups of older adults with and without cognitive impairment. Such data are fundamental to document the development of reduced manual asymmetry in old age, which most likely represents a behavioral adaptation to normal neurophysiological constraints caused by the wear and tear of the neuromuscular system, rather than being solely the result of pathology. However, conditions like MCI that are superimposed upon normal senescence processes appear to accelerate the development of symmetric hand performance.

From a clinical point of view these findings are highly relevant in light of the new ICD-11 6D71 “Mild neurocognitive disorder” (World Health Organization, 2022), which shifts from the previous ICD-10 F06.7 framework regarding how disability is defined, specifically concerning Instrumental Activities of Daily Living (IADLs). ICD-10 F06.7 focused on a general, often subjective decline in cognitive performance. In contrast, ICD-11 6D71 explicitly defines the disorder by the preservation of independent everyday functioning. Because the new ICD-11 6D71 places a stronger clinical emphasis on objective evidence, it opens the door for the closer integration of fine motor function, specifically hand dexterity, as a quantifiable marker of functional ability and cognitive-motor integration in everyday life. Therefore, the early emergence of symmetric hand function may serve as a highly accessible behavioral marker for the early diagnosis of neurodegenerative diseases.

## Data Availability

The datasets for this article cannot be made publicly available due to restrictions of the Norwegian Research Ethics Committee. However, upon reasonable request, summary statistics can be obtained from the corresponding author (CRA). Requests to access the datasets should be directed to claudia.rodriguez-aranda@uit.no.
