# Supplementary material for: Longitudinal changes in hand dominance and manual dexterity in normal aging and mild cognitive impairment (MCI): a kinematic study
Source: Front Aging Neurosci. 2026 Oct 2;18:1921208. doi: 10.3389/fnagi.2026.1921208 (PMC13672655; doi:10.3389/fnagi.2026.1921208)
Supplement: Supplementary file 1 [file Data_Sheet_1.DOCX]

**Supplementary Figures and Tables**

**Supplementary Figure 1.** Individual trajectories of laterality indices during reaching in the healthy control group (n = 20). T1 = first test. T2 = retest. LinV = linear velocity (cm/s). CV = coefficient of variation (SD/M). PL = path length (cm). Only LIs from kinematic parameters showing significant changes from T1 to T2 in the main analysis are depicted.

**Supplementary Figure 2.** Individual trajectories of laterality indices during grasping in the healthy control group (n = 20). T1 = first test. T2 = retest. LinV = linear velocity (cm/s). CV = coefficient of variation (SD/M). PL = path length (cm). Only LIs from kinematic parameters showing significant changes from T1 to T2 in the main analysis are depicted.

Supplementary Table 1. Multivariate effects of all factors by movement type.

Reaching	Grasping	Transport	Inserting

F	η^2^_p_	F	η^2^_p_	F	η^2^_p_	F	η^2^_p_

Laterality indices

Time	4.02**	.501	7.0***	.636	2.66*	.399	3.48**	.465

Group	4.27**	.516	9.83***	.711	3.26*	.449	3.43**	.461

Time × Group	7.66***	.657	7.55***	.654	8.32***	.675	4.22**	.513

Kinematics

Time	15.91***	.799	12.90***	.763	17.64***	.815	7.96***	.665

Group	9.85***	.711	4.28**	.517	23.69***	.856	11.59***	.743

Hand	17.80***	.817	19.39***	.829	26.82***	.870	7.48***	.651

Time × Group	38.79***	.907	22.44***	.849	30.17***	.883	18.98***	.826

Hand × Group	5.01***	.556	5.96***	.598	5.16***	.563	3.39**	.459

Time × Hand	4.76**	.543	6.77***	.629	4.05**	.503	3.36*	.456

Time × Hand × Group	8.58***	.682	3.20*	.444	9.27***	.699	4.66**	.538

Note. Degrees of freedom for all effects are 7, 28. *** = p <.001. ** = p <.01. * = p <.05. F is equal to Pillai’s V.

Supplementary Table 2. Multivariate effects of all factors by movement type, with time interval between assessments (Δ t) as covariate.

Reaching	Grasping	Transport	Inserting

F	η^2^_p_	F	η^2^_p_	F	η^2^_p_	F	η^2^_p_

Laterality indices

Time	2.08	.398	6.28***	.666	2.51*	.444	2.53*	.446

Group	.81	.206	2.66*	.459	2.12	.403	1.70	.351

Time × Group	2.40*	.433	3.73**	.543	3.49*	.526	1.80	.364

Δ t	1.12	.232	.68	.156	.97	.207	1.18	.241

Δ t × Time	1.15	.236	1.68*	.311	1.02	.215	1.22	.247

Δ t × Group	1.09	.257	1.08	.257	1.08	.256	.97	.235

Δ t × Time × Group	.96	.234	.75	.193	.29	.086	2.22*	.414

Kinematics

Time	8.73**	.735	5.30**	.628	7.12***	.694	5.99***	.656

Group	3.43*	.522	2.68*	.460	6.44***	.672	5.69***	.644

Hand	7.61***	.708	10.13***	.763	9.96***	.760	2.13	.404

Time × Group	9.10***	.743	6.21***	.664	8.62***	.733	5.06**	.617

Hand × Group	.85	.213	1.63	.341	2.07	.347	1.34	.299

Time × Hand	2.49*	.442	4.15**	.569	3.96**	.557	2.51*	.444

Time × Hand × Group	2.61*	.454	2.50*	.443	4.17**	.570	1.88	.375

Δ t	1.25	.252	1.39	.272	1.50	.288	1.10	.228

Δ t × Time	1.39	.273	1.48	.285	.87	.190	1.22	.246

Δ t × Group	1.03	.247	.44	.122	.38	.108	.49	.134

Δ t × Time × Group	.90	.222	.94	.231	.48	.133	.32	.094

Δ t × Hand	1.16	.238	.80	.178	.92	.199	1.08	.225

Δ t × Hand × Group	.85	.213	.82	.207	.40	.113	.88	.218

Δ t × Time × Hand	1.05	.220	1.52*	.291	1.02	.215	1.47	.283

Δ t × Time × Hand × Group	.74	.190	.55	.149	.24	.071	2.16	.407

Note. Degrees of freedom for all effects are 7, 27. *** = p <.001. ** = p <.01. * = p <.05. F is equal to Pillai’s V.

Supplementary Table 3. Longitudinal changes in laterality indices. Results of pairwise comparisons.

Reaching	Grasping

HC	MCI	HC	MCI

T1	T2	t	95% CI	T1	T2	t	95% CI	T1	T2	t	95% CI	T1	T2	t	95% CI

LinV	**.11	.01	7.21*** [.07; .13]**	.01	.03	-1.25	[-.05; .01]	**.14	.06	2.89**	[.02; .14]**	.05	.04	.58	[-.05; .08]

CV linV **-.20 -.04	-5.09*** [-.23; -.10]**	-.03 -.04	.51	[-.05; .09]	**.12	-.01 5.08*** [.08; .18]**	.01	-.01	.45	[-.05; .07]

PL	**-.07 -.03	-2.76** [-.08; -.01]**	.01	-.01	.47	[-.03; .05]	**.24	.07 4.07*** [.09; .26]**	.04	.03	.11	[-.09; .10]

AngV	.05 -.03	1.49	[-.03; .20]	.08	.01	1.13 [-.06; .20]	.16	.09 1.42	[-.03; .17]	.13	.07	1.0	[-.06; .17]

CV angV	.02	.10	-1.47	[-.19; .03]	-.01	.01	-.27	[-.14; .11]	.01	.02 -.32	[-.08; .06]	.03	-.02 1.24 [-.03; .12]

Angle	**.11	-.01	2.60*	[.03; .20]**	.04	.08	-.79	[-.14; .06]	**.16	.03	2.70*	[.03; .22]**	.06	.12 -1.11 [-.17; .05]

CV angle **-.23 -.05 -2.49*	[-.32; -.03]**	-.13 -.18	2.01	[-.01; .32]	.02	.01	.21	[-.13; .16]	.05	-.11	1.94 [-.01; .32]

Transport	Inserting

HC	MCI	HC	MCI

T1	T2	t	95% CI	T1	T2	t	95% CI	T1	T2	t	95 % CI	T1	T2	t	95% CI

LinV	**.13	.06	4.0***	[.04; .11]**	.04	.07	-1.38	[-.07; .01]	**.10	.02	2.81**	[.02; .13]**	-.02	.01 -.66	[-.08; .04]

CV linV	**-.23	-.10 -4.19*** [-.20; -.07]**	-.06 -.06	.03	[-.07; .07]	**.08	.02	3.69*** [.03; .09]**	.02	.01	.33 [-.03; .04]

PL	**-.08	-.01 -3.17**	[-.13; -.03]**	-.02 -.02	-.15	[-.06; .05]	**.10	-.01 3.68*** [.05; .18]**	-.11	-.07 -1.15 [-.11; .03]

Ang V	.04	.04	-.74	[-.18; .08]	**.15	-.04	2.53* [.04; .33]**	.15	.12	.66	[-.06; .11]	.01	.04 -.60 [-.12; .07]

CV angV **-.06	.06	2.65*	[-.23; -.13]**	.04	-.02	1.19	[-.05; .17]	.01	.01	.09	[-.07; .07]	-.03 -.03	.05 [-.08; .08]

Angle	.10	.01	2.20	[.01; .17]	.04	.04	.17	[-.09; .10]	.07	-.01	2.19	[.01; .15]	.02	.01	.35 [-.07; .10]

CV angle	**-.27 .02	-3.24**	[-.46; -.11]**	-.01	-.10	.98	[-.10; .29]	.04	.01	.42	[-.11; .16]	-.05	.01 -.78 [-.21; .09]

*Note.* HC = healthy controls. MCI = mild cognitive impairment. T1 = first test. T2 = retest. LinV = linear velocity (cm/s). CV = coefficient of variation (SD/M). PL = path length (cm). AngV = angular velocity (°/s).

Supplementary Table 4. Longitudinal changes in laterality indices of reaching and grasping with time interval as covariate. Results of pairwise comparisons.

Reaching	Grasping

HC	MCI	HC	MCI

T1	T2	t	95% CI	T1	T2	t	95% CI	T1	T2	t	95% CI	T1	T2	t	95% CI

LinV	**.11	.01	5.90*** [.07; .14]**	.01	.03	-1.0	[-.06; .02]	**.16	.06	3.12**	[.11; .21]**	.03	.04	.26	[-.03; .10]

CV linV **-.19 -.01	-4.70*** [-.26; -.10]**	-.03 -.07	.86	[-.05; .13]	**.12	-.01 4.29*** [.09; .16]**	.01	-.01	.33	[-.03; .05]

PL	**-.07 -.02	-2.45*	[-.09; -.01]**	.01	-.01	.46	[-.04; .06]	**.28	.05 4.73*** [.22; .33]**	-.01	.06 -1.16 [-.07; .06]

AngV	.04 -.02	.87	[-.08; .20]	.09	-.01	1.29 [-.06; .27]	.15	.10	.88	[.06; .24]	.14	.06	1.13 [.04; .25]

CV angV	.02	.11	-1.38	[-.22; .04]	-.01	-.01	-.13	[-.15; .15]	.04	-.01 1.27	[-.01; .08]	-.01	.02	-.56 [-.06; .05]

Angle	**.11	-.05	3.20**	[.06; .26]**	.04	.14	-1.7	[-.22; .02]	**.20	.03 2.98**	[.08; .31]**	.02	.13 -1.68 [-.11; .15]

CV angle **-.23 -.01 -2.70*	[-.40; -.05]**	**-.02 -.23	2.26* [.02; .42]**	.02	-.01	.37	[-.13; .17]	.05	-.08	1.31[-.12; .23]

*Note.* HC = healthy controls. MCI = mild cognitive impairment. T1 = first test. T2 = retest. LinV = linear velocity (cm/s). CV = coefficient of variation (SD/M). PL = path length (cm). AngV = angular velocity (°/s).

Supplementary Table 5. Univariate effects of all factors by movement type.

LinV	CV linV	PL	AngV	CV angV	Angle	CV angle	F	η^2^_p_	F	η^2^_p_	F	η^2^_p_	F	η^2^_p_	F	η^2^_p_	F	η^2^_p_	F	η^2^_p_

**Laterality indices**

Reaching

Time	14.29**	.296	9.34**	.215	2.28	.063	3.35	.090	1.40	.039	1.31	.037	.03	.001

Group	8.42**	.198	13.34*** .282	9.86**	.225	.67	.019	1.96	.054	.07	.002	.28	.008

Time × Group	32.41*** .488	14.48*** .299	5.05*	.129	.03	.001	.61	.018	5.46*	.138	9.94**	.226

Grasping

Time	5.57*	.141	14.08*** .293	7.73**	.185	2.91	.079	.53	.015	.90	.348	2.48	.068

Group	4.58*	.119	19.53*** .365	13.96*** .291	3.45	.010	.06	.002	.01	.001	.39	.011

Time × Group	2.25	.062	9.51**	.219	6.85*	.168	.04	.001	1.34	.038	6.88*	.168	1.70	.048

Transport

Time	2.44	.067	8.05**	.191	4.85*	.125	1.96	.054	.75	.022	2.51	.069	2.06	.057

Group	4.32*	.113	20.55*** .377	1.86	.052	.03	.001	.09	.003	.26	.008	.97	.028

Time × Group	13.51*** .284	8.01**	.191	3.86	.102	5.70*	.144	6.86*	.168	1.74	.099	8.43	.199

Inserting

Time	1.92	.053	7.55*	.182	2.65	.072	.01	.001	.01	.001	2.96	.080	.09	.003

Group	7.82**	.187	3.0	.081	14.46*** .298	5.90*	.148	1.92	.053	.75	.021	.76	.022

Time × Group	5.65*	.142	5.07*	.130	11.15**	.247	.79	.023	.01	.001	1.44	.090	.73	.022

**Kinematics**

Reaching

Time	.09	.003	21.43*** .387	32.51*** .489	4.25*	.111	9.72** .222	.01	.001	.06	.002	Group	3.80	.100	4.02	.106	14.41*** .298	.65	.019	40.31*** .542	.02	.001	9.96**	.227

Hand	21.70*** .390	51.23*** .601	16.33*** .324	1.92	.053	4.49*	.117	8.79**	.205	15.60*** .314

Time × Group	25.80*** .431	52.09*** .601	26.76*** .440	10.74** .240	8.55**	.201	9.91**	.226	26.08*** .434

Hand × Group	7.82**	.187	17.50*** .340	11.17**	.247	.71	.021	2.48	.068	.14	.004	.01	.001

Time × Hand	11.09**	.246	15.09*** .307	3.98	.105	5.23*	.133	1.79	.050	1.10	.031	.09	.003

Time × Hand × Group 25.13*** .425	26.28*** .436	7.38*	.178	.55	.016	.26	.007	5.21*	.133	13.20*** .289

Grasping

Time	2.25	.062	25.16*** .425	5.96**	.149	.75	.021	.37	.011	.37	.011	1.84	.051

Group	8.10**	.193	11.31**	.250	.03	.001	.72	.021	.66	.019	.53	.015	12.10** .262

Hand	30.71*** .475	13.22*** .280	24.14*** .415	21.23*** .334	1.0	.029	11.26** .249	.64	.018

Time × Group	35.27*** .509	16.96*** .333	21.09*** .383	6.99*	.171	17.99*** .346	5.29*	.134	2.23	.062

Hand × Group	4.13	.108	12.21*** .264	8.30**	.196	.04	.001	.23	.007	.01	.001	.27	.008

Time × Hand	3.87	.102	11.23**	.248	3.41	.091	2.41	.066	.52	.015	1.64	.046	.44	.013

Time × Hand × Group	.24	.007	6.88*	.168	1.71	.048	.27	.008	1.91	.053	4.59*	.119	.04	.001

Transport

Time	.78	.022	27.11**	.444	31.95*** .484	18.87*** .357	11.79**	.258	.01	.001	1.68	.047

Group	7.53*	.181	1.13	.032	21.72*** .390	1.01	.029	60.10*** .639	.11	.013	4.77*	.123

Hand	60.55*** .668	85.51*** .716	11.80**	.258	4.95*	.127	.01	.001	14.21*** .295	3.28	.088

Time × Group	15.34*** .311	81.59*** .706	18.05*** .347	.01	.001	19.68*** .366	14.32*** .296	20.87*** .380	Hand × Group	9.26**	.214	21.60*** .388	3.42	.091	.11	.003	.40	.012	.14	.004	1.75	.049

Time × Hand	3.29	.088	15.57*** .314	6.06*	.151	6.42*	.159	1.90	.053	2.38	.065	2.60	.071

Time × Hand × Group	7.76**	.186	19.96*** .370	5.17*	.132	4.94*	.127	8.83**	.195	1.70	.048	5.01*	.128

Inserting

Time	.11	.003	13.17*** .279	.54	.016	2.86	.078	8.93**	.208	.44	.013	2.24	.062

Group	3.45	.092	.58	.017	.03	.001	.18	.005	21.00*** .382	1.10	.031	4.22*	.110

Hand	5.05*	.129	10.40**	.234	1.05	.030	13.82**	.289	.44	.013	2.87	.078	.03	.001

Time × Group	5.86*	.147	39.76*** .539	1.72	.198	3.82	.101	8.76**	.205	20.02*** .371	3.52	.094

Hand × Group	8.65**	.203	2.31	.064	13.28*** .181	7.13*	.173	1.11	.032	.48	.014	.10	.003

Time × Hand	1.76	.049	7.09*	.173	1.84	.051	.05	.001	.01	.001	2.31	.064	.30	.009

Time × Hand × Group	5.15*	.132	3.90	.103	9.84**	.225	1.69	.047	.01	.001	1.03	.029	.52	.015

_______________________________________________________________________________________________________________________________________________________________________________________________________________________________________________________________________________________

Note. Degrees of freedom for all effects are 1, 34. *** = p <.001. ** = p <.01. * = p <.05.

Supplementary Table 6. Absolute values of laterality indices by movement type.

Reaching	Grasping

HC	MCI	HC	MCI

T1 (M(SD))	T2 (M(SD))	T1 (M(SD))	T2 (M(SD))	T1 (M(SD))	T2 (M(SD))	T1 (M(SD))	T2 (M(SD))

LinV	.11(.05)	.04(.03)	.04(.03)	.06(.04)	.15(.08)	.09(.08)	.10(.05)	.09(.07)

CV linV	.20(.08)	.09(.06)	.07(.05)	.10(.08)	.13(.07)	.06(.04)	.04(.03)	.05(.05)

PL	.08(.05)	.06(.04)	.05(.03)	.04(.03)	.24(.11)	.12(.10)	.09(.07)	.15(.11)

AngV	.14(.09)	.13(.11)	.14(.10)	.19(.16)	.17(.13)	.16(.12)	.20(.10)	.22(.13)

CV angV	.08(.06)	.12(.12)	.17(.13)	.14(.14)	.07(.06)	.09(.08)	.07(.05)	.07(.06)

Angle	.14(.11)	.10(.10)	.12(.10)	.13(.07)	.23(.17)	.11(.11)	.15(.09)	.16(.14)

CV angle	.25(.21)	.23(.11)	.20(.14)	.23(.15)	.20(.15)	.20(.15)	.25(.15)	.26(.16)

Transport	Inserting

HC	MCI	HC	MCI

T1 (M(SD))	T2 (M(SD))	T1 (M(SD))	T2 (M(SD))	T1 (M(SD))	T2 (M(SD))	T1 (M(SD))	T2 (M(SD))

LinV	.13(.05)	.07(.05)	.07(.04)	.10(.06)	.10(.06)	.06(.05)	.06(.04)	.10(.11)

CV linV	.23(.06)	.11(.08)	.11(.06)	.10(.09)	.09(.07)	.05(.04)	.06(.03)	.03(.02)

PL	.10(.06)	.07(.05)	.06(.05)	.05(.04)	.13(.12)	.10(.08)	.13(.07)	.11(.07)

Ang V	.14(.11)	.21(.15)	.18(.15)	.14(.11)	.17(.11)	.16(.12)	.17(.11)	.12(.07)

CV angV	.09(.07)	.14(.14)	.14(.13)	.12(.07)	.08(.07)	.08(.06)	.10(.06)	.07(.06)

Angle	.13(.07)	.10(.11)	.09(.06)	.11(.13)	.11(.07)	.10(.09)	.08(.05)	.09(.09)

CV angle	.31(.16)	.26(.21)	.24(.17)	.24(.21)	.15(.13)	.20(.15)	.21(.11)	.12(.12)

*Note.* HC = healthy controls. MCI = mild cognitive impairment. T1 = first test. T2 = retest. M(SD) represent raw means and standard deviations. LinV = linear velocity (cm/s). CV = coefficient of variation (SD/M). PL = path length (cm). AngV = angular velocity (°/s).

Supplementary Table 7. Means and standard deviations of kinematic parameters by movement type and session.

Reaching

_________________HC_______________________	__________________MCI________________________

R T1	R T2	L T1	L T2	R T1	R T2	L T1	L T2

LinV	46.3(7.4)	49.6(7.7)	36.9(4.5)	48.5(6.9)	44.9(8.2)	39.19(9.2)	44.5(9.3)	37.0(8.6)

CV linV	.32(.05)	.23(.04)	.48(.07)	.25(.04)	.26(.05)	.32(.05)	.27(.04)	.35(.08)

PL	138.9(15.3)	117.8(12.6)	160.4(15.7)	124.0(14.4)	121.0(13.5)	118.4(16.8)	121.1(18.2)	120.9(21.1)

AngV	72.7(21.6)	48.6(12.7)	63.9(12.4)	54.6(20.5)	59.9(20.1)	59.9(20.8)	50.3(14.3)	57.9(18.1)

CV angV	.70(.12)	.60(.09)	.67(.09)	.50(.12)	.45(.15)	.46(.17)	.45(.16)	.43(.13)

Angle	45.0(11.3)	46.3(11.5)	36.4(10.8)	46.5(10.4)	47.9(10.1)	43.9(12.4)	44.2(10.3)	36.9(8.6)

CV angle	.13(.05)	.11(.09)	.20(.07)	.11(.05)	.15(.09)	.18(.07)	.16(.08)	.25(.06)

Grasping

_________________HC_______________________	__________________MCI________________________

R T1	R T2	L T1	L T2	R T1	R T2	L T1	L T2

LinV	8.6(1.6)	10.9(2.1)	6.4(1.2)	9.6(2.1)	9.2(2.4)	7.2(1.7)	8.1(1.3)	6.7(1.5)

CV linV	.67(.07)	.72(.10)	.52(.08)	.73(.09)	.72(.09)	.73(.10)	.71(.08)	.74(.09)

PL	80.4(22.6)	98.3(22.6)	48.9(15.4)	84.6(19.6)	85.0(27.3)	75.2(25.8)	77.5(20.0)	70.9(28.5)

AngV	57.3(20.5)	58.1(17.1)	40.0(12.2)	49.6(19.0)	67.8(19.0)	55.5(28.0)	51.5(12.2)	43.6(11.8)

CV angV	.75(.10)	.68(.13)	.74(.08)	.65(.08)	.68(.11)	.7(.14)	.64(.09)	.73(.13)

Angle	43.4(15.6)	46.0(12.0)	31.7(13.8)	43.8(14.8)	43.7(15.7)	40.7(14.5)	38.3(12.2)	32.8(15.5)

CV angle	.20(.09)	.26(.14)	.20(.11)	.28(.17)	.33(.15)	.32(.18)	.35(.13)	.36(.16)

Transport

_________________HC_______________________	__________________MCI________________________

R T1	R T2	L T1	L T2	R T1	R T2	L T1	L T2

LinV	35.7(4.6)	36.4(6.7)	27.4(4.3)	32.2(5.1)	32.2(5.0)	28.4(6.2)	29.8(5.5)	25.1(6.8)

CV linV	.28(.03)	.23(.04)	.45(.04)	.28(.04)	.27(.05)	.30(.04)	.30(.06)	.33(.08)

PL	108.3(14.7)	92.2(11.1)	127.9(19.5)	93.9(15.0)	91.4(13.4)	88.2(11.3)	95.0(14.0)	91.1(13.5)

AngV	62.4(21.5)	47.9(18.3)	56.5(18.0)	43.6(22.9)	73.6(22.1)	47.3(13.3)	54.2(19.2)	52.9(19.3)

CV angV	.73(.12)	.65(.12)	.82(.09)	.58(.15)	.51(.14)	.50(.12)	.47(.15)	.52(.11)

Angle	58.4(10.2)	61.0(12.3)	47.5(7.4)	59.2(10.3)	63.6(12.2)	56.1(13.4)	58.0(10.9)	51.2(9.2)

CV angle	.09(.05)	.07(.05)	.16(.07)	.07(.05)	.11(.06)	.14(.10)	.11(.06)	.15(.07)

Inserting

_________________HC_______________________	__________________MCI________________________

R T1	R T2	L T1	L T2	R T1	R T2	L T1	L T2

LinV	7.8(1.5)	8.1(1.4)	6.5(1.2)	7.7(1.5)	6.93(1.5)	6.5(1.8)	7.18(1.7)	6.5(1.8)

CV linV	.71(.08)	.80(.08)	.62(.08)	.78(.10)	.77(.09)	.73(.08)	.75(.07)	.72(.07)

PL	75.9(29.0)	74.2(21.3)	60.5(18.0)	75.4(16.6)	63.5(18.3)	64.9(20.7)	79.3(24.2)	74.2(21.6)

AngV	54.8(16.5)	44.2(16.4)	41.1(15.9)	34.9(13.5)	41.9(13.3)	44.1(16.2)	41.6(15.4)	40.7(13.8)

CV angV	.91(.12)	.79(.14)	.91(.17)	.78(.12)	.68(.17)	.68(.14)	.71(.15)	.71(.12)	Angle	48.5(11.0)	52.3(11.8)	41.9(10.7)	52.4(10.6)	57.3(12.1)	47.0(11.2)	55.3(11.1)	46.3(9.3)

CV angle	.23(.09)	.22(.11)	.22(.10)	.21(.11)	.24(.12)	.32(.19)	.26(.11)	.31(.13)

Note. HC = healthy controls. MCI = mild cognitive impairment. R = right hand. L= left hand. T1 = first test session. T2 = retest session. LinV = linear velocity (cm/s). CV = coefficient of variability (SD/M). PL = path length (cm). AngV = angular velocity (°/s
